# Estrogen Augments Shear Stress–Induced Signaling and Gene Expression in Osteoblast-like Cells via Estrogen Receptor–Mediated Expression of β_1_-Integrin

**DOI:** 10.1359/jbmr.091008

**Published:** 2009-10-12

**Authors:** Chiuan-Ren Yeh, Jeng-Jiann Chiu, Chih-I Lee, Pei-Ling Lee, Yu-Tsung Shih, Jui-Sheng Sun, Shu Chien, Cheng-Kung Cheng

**Affiliations:** 1 Institute of Biomedical Engineering, National Yang-Ming UniversityTaiwan, ROC; 2 Division of Medical Engineering Research, National Health Research InstitutesTaiwan, ROC; 3 Department of Orthopaedic Surgery, National Taiwan University HospitalTaiwan, ROC; 4 Departments of Bioengineering and Medicine and Institute of Engineering in Medicine, University of CaliforniaSan Diego, La Jolla, CA, USA

**Keywords:** osteoblast, gene expression, estrogen, integrin, mapk, shear stress

## Abstract

Estrogen and mechanical forces are positive regulators for osteoblast proliferation and bone formation. We investigated the synergistic effect of estrogen and flow-induced shear stress on signal transduction and gene expression in human osetoblast-like MG63 cells and primary osteoblasts (HOBs) using activations of extracellular signal-regulated kinase (ERK) and p38 mitogen-activated protein kinase (MAPK) and expressions of *c-fos* and cyclooxygenase-2 (I) as readouts. Estrogen (17β-estradiol, 10 nM) and shear stress (12 dyn/cm^2^) alone induced transient phosphorylations of ERK and p38 MAPK in MG63 cells. Pretreating MG63 cells with 17β-estradiol for 6 hours before shearing augmented these shear-induced MAPK phosphorylations. Western blot and flow cytometric analyses showed that treating MG63 cells with 17β-estradiol for 6 hrs induced their β_1_-integrin expression. This estrogen-induction of β_1_-integrin was inhibited by pretreating the cells with a specific antagonist of estrogen receptor ICI 182,780. Both 17β-estradiol and shear stress alone induced *c-fos* and *Cox-2* gene expressions in MG63 cells. Pretreating MG63 cells with 17β-estradiol for 6 hrs augmented the shear-induced *c-fos* and *Cox-2* expressions. The augmented effects of 17β-estradiol on shear-induced MAPK phosphorylations and *c-fos* and *Cox-2* expressions were inhibited by pretreating the cells with ICI 182,780 or transfecting the cells with β_1_-specific small interfering RNA. Similar results on the augmented effect of estrogen on shear-induced signaling and gene expression were obtained with HOBs. Our findings provide insights into the mechanism by which estrogen augments shear stress responsiveness of signal transduction and gene expression in bone cells via estrogen receptor–mediated increases in β_1_-integrin expression. © 2010 American Society for Bone and Mineral Research.

## Introduction

Mechanical loading is essential for maintaining skeletal integrity and bone mass.(1) Suppression of this stimulus under conditions such as long-term bed rest and space flight results in bone loss and even osteoporosis.(2) During dynamic loading of intact bone, interstitial fluid (ISF) flow through the canaliculi generates shear stress that is detected by bone cells, including osteoblasts.(3) It has been shown that fluid shear stress regulates signaling, gene expression, proliferation, and differentiation in osteoblasts.(4) Recent studies using flow channels demonstrated that application of fluid shear stress to osteoblasts induces their expression of many genes, including transcription factor *c-fos*^5^ and cyclooxygenase-2 (*Cox-2*),(5) both of which have been shown to play important roles in bone formation in vivo.(6,7)

In addition to mechanical forces, estrogen, a major sex steroid hormone in female, has been shown to induce osteoblast proliferation and bone formation, with suppression in bone tissue resorption by decreasing osteoclast activity.(8) A deficiency of estrogen accelerates bone lose; this is probably the major reason leading to osteoporosis in menopausal women.(9) 17β-Estradiol represents a major estrogen in human, and it is produced by granulose cells of the ovary, with small amounts also being produced by the adrenal cortex. Recent studies showed that 17β-estradiol induces insulin-like growth factor 1 (IGF-1) and alkaline phosphatase (ALP) production through estrogen receptors (ERs) to increase osteoblast proliferation and bone formation.(10,11) Blockage of ERs by the specific ER antagonist ICI 182,780 inhibited 17β-estradiol-induced IGF-1 and ALP productions by osteoblasts.(10,11) In addition, estrogen has been shown to inhibit osteoblast and osteocyte apoptosis and promote the survival of these cells, which contributes to the beneficial effect of the steroids in bone.(12–14) Although both estrogen and mechanical force have been shown to be positive regulators for osteoblast proliferation and bone formation, whether they play synergistic roles in modulating signaling and gene expression in osteoblasts remains unclear.

Integrins, as the main receptors that connect the cytoskeleton and the extracellular matrix (ECM), have been shown to play important roles in transmitting mechanical stresses into chemical signals in a wide variety of cells seeded on the ECM.(15) Human osteoblasts express several types of integrins, including those containing the β_1_ subunit (dimerized with α subunits, including α_1_, α_3_, α_5_, and α_6_),(16) which have been shown to play important roles in osteoblast differentiation and commitment.(17) In several systems, including endothelial cells, shear stress activation of integrins leads to an increase in their association with an adaptor protein, Shc, that subsequently activates the downstream mitogen-activated protein kinases (MAPKs), including extracellular signal-regulated kinase (ERK) and p38 MAPK.(18) Recent studies demonstrated that estrogen induces β_1_-integrin expression and adhesive properties in endothelial cells.(19) Although integrins have been well recognized as mechanosensors in a variety of cells in response to mechanical stimuli, whether estrogen regulates mechanical responsiveness of signaling and gene expression in osteoblasts by modulating their integrin expression remains unclear.

In this study, we investigated the synergistic role of estrogen and shear stress in signaling and gene expression in both human osteoblast-like MG63 cells and primary osteoblasts (HOBs) using activations of ERK and p38 MAPK and expressions of *c-fos* and *Cox-2* as readouts. MG63 cells were derived originally from an osteogenic sarcoma of a 14-year-old boy and exhibit many osteoblast traits characteristic of bone-forming cells.(20) Our results showed that pretreatment of MG63 cells and HOBs with 17β-estradiol enhances shear stress–induced activations of ERK and p38 MAPK and expressions of *c-fos* and *Cox-2* through ER-mediated increases in β_1_-integrin expression. Our findings provide insights into the mechanism by which estrogen augments shear stress responsiveness of signal transduction and bone-formation-related gene expression in bone cells via ER-mediated induction of β_1_-integrin.

## Materials and Methods

### Materials

Mouse monoclonal antibodies (mAbs) against ERK2 (sc-1647) and phospho-ERK1/2 (sc-7383) were purchased from Santa Cruz Biotechnology (Santa Cruz, CA, USA). Rabbit polyclonal antibody (pAb) against p38 MAPK and mouse mAb against phospho-p38 MAPK were purchased from Cell Signaling Technology (Beverly, MA, USA). Mouse mAb against human β_1_-integrin was purchased from Chemicon (MAB 2253, Temecula, CA, USA). The phycoerythrin (PE)–conjugated mouse anti-human CD29 (β_1_-integrin chain) antibody was purchased from BD Biosciences (San Jose, CA, USA). The control small interfering RNA (siRNA) and specific siRNA of β_1_-integrin were purchased from Invitrogen (Carlsbad, CA, USA). The type I collagen, charcoal (dextran coated), and 17β-estradiol were obtained from Sigma (St. Louis, MO, USA). The ICI 182,780 was purchased from Torics (Ellisville, MO, USA). All other chemicals of reagent grade were obtained from Sigma unless otherwise noted.

### Cell culture

The human osteoblast-like MG63 cells were obtained from American Type Culture Collection (Rockville, MD, USA) and cultured in a phenol red–free medium (α-MEM, Gibco, Gaithersburg, MD, USA) supplemented with 10% fetal bovine serum (FBS, Gibco). Human bone specimens were collected aseptically during orthopedic surgery of the knee or the hip, and the primary HOBs were harvested and cultured by using the method described previously.(10) Osteocalcin expression and alkaline phosphatase activity were examined to confirm the osteoblastic phenotype of HOBs. The HOBs were cultured in a phenol red–free medium (DMEM, Gibco) supplemented with 20% FBS. After reaching confluence, these cells were trypsinized and seeded onto glass slides (75 × 38 mm, Corning Incorporated, Corning, NY, USA) that had been precoated with type I collagen (30 µg/mL) at a concentration of 10^4^ cells/cm^2^. The cells were incubated in phenol red–free α-MEM supplemented with 10% FBS (pretreated with charcoal to remove endogenous estrogen in the serum) for 24 hrs. The medium then was changed to phenol red– and serum-free α-MEM containing 0.25% bovine serum albumin (BSA) for incubating the cells for 48 hrs prior to the experiments.

### Flow apparatus

The slide with cultured MG63 cells or HOBs was mounted in a parallel-plate flow chamber, which has been characterized and described in detail elsewhere.(21) The chamber was connected to a perfusion loop system, kept in a constant temperature-controlled enclosure, and maintained at pH 7.4 by continuous gassing with humidified 5% CO_2_ in air. The flow channel width (*w*) was 1 cm, and the channel height (*h*) was 0.025 cm. The fluid shear stress (τ) generated on the cells by flow was calculated to be 12 dyn/cm^2^, using the formula τ = 6µ*Q*/*wh*^2^, where µ is the dynamic viscosity of the perfusate and *Q* is the flow rate. In some experiments, MG63 cells or HOBs were incubated with the specific inhibitor for ERs (ICI 182,780, 10 nM), ERK (PD98059, 30 µM), or p38 MAPK (SB203580, 10 µM) for 1 hr before stimulation with 17β-estradiol (10 nM) or exposure to flow.

### Western blot analysis

The cells were lysed with a buffer containing 1% NP-40, 0.5% sodium deoxycholate, 0.1% SDS, and a protease inhibitor mixture (PMSF, aprotinin, and sodium orthovanadate). The total cell lysate (100 µg of protein) was separated by SDS-PAGE (12% running, 4% stacking) and analyzed by using the designated antibodies and the Western-Light chemiluminescent detection system (Applied Biosystems, Foster City, CA, USA), as described previously.(21)

### RNA isolation and reverse transcriptase-polymerase chain reaction (RT-PCR)

Total RNA was isolated by the guanidium isothiocyanate/phenochloroform method and converted to cDNA, as described previously.(21) RT-PCR analysis was performed with the RT System (Promega, San Luis Obispo, CA, USA) according to the manufacturer's protocols. Briefly, the RT reaction with 2 µg of total RNA was performed with the following cycles: 65°C for 5 minutes, 42°C for 50 minutes, 70°C for 15 minutes, and 37°C for 20 minutes. For each reaction, cDNA from the RT reaction was amplified by PCR with the use of 2.5 units of Taq DNA polymerase (Promega) and the β_1_-integrin primers, as shown in Table 1. The PCR reactions were carried out in a GeneAmps System 9700 (PE Biosystems, Foster City, CA, USA). The PCR cycles for each reaction were as follows: heat denaturation at 94°C for 30 seconds, primer annealing at 60°C for 30 seconds, and primer extension at 72°C for 45 seconds. The amplified cDNAs were analyzed by 1% agarose gel electrophoresis and ethidium bromide staining. Band intensities were quantified from the stained agarose gels using video imaging and a densitometry software system (GDS-8000 Imaging Workstation, UVP, Inc., Upland, CA, USA).

**Table 1:** Primer Sequences and the Number of Reaction Cycles Used for RT-PCR

Gene name	Gene bank accession number	Primer sequence	Size (bp)	Number of cycles
*c-fos*	V01512	F-TGCTTTCAGACTGGGCTCTT	73	45
		R-GCAGAATAGGTGTGACTTGCAT		
*Cyclooxygenase-2*	U04636	F-TCACGCATCAGTTTTTCAAGA	94	45
*(Cox-2)*		R-TCACCGTAAATATGATTTAAGTCCAC		
*β-actin*	NM001101	F-AAATCGTCCGTGACATCAAG	180	45
		R-GGAAGGAAGGCTGG AAGA GA		
*β*_*1*_*-integrin*	NM002211	F-ACAGAAGAAGTAGAGGTGGTC	660	30
		R-GAGGTTGAAATGGGAGG		
*GAPDH*	AF261085	F-CCACCCATGGCAAATTCCATGGCA	599	27
		R-TCTAGACGGCAGGTCAGGTCCACC		

### Quantitative real-time PCR

The cDNA was amplified through PCR on a LightCycler (Roche Diagnostics, East Sussex, United Kingdom) using LightCycler FastStart DNA MasterPlus SYBR Green I (Roche Diagnostics) with 0.5 µM primers of *c-fos* and *Cox-2* genes (see Table 1). PCR was performed in triplicate at 95°C for 10 minutes, followed by 45 cycles of denaturation at 95°C for 10 seconds, annealing at 60°C for 5 seconds, extension at 72°C for 8 seconds, and single signal acquisition for 10 seconds. The β-actin gene expression was used as an internal control. The PCR conditions were optimized to obtain a PCR product with a single peak on melting-curve analysis on the LightCycler. Raw data collected from the LightCycler were analyzed using LightCycler Software Version 3.5 (Roche Diagnostics). The *c-fos* and *Cox-2* gene expression levels were normalized with β-actin gene expression level in the same sample.

### Flow cytometric analysis

The cells were harvested in PBS containing 2 mM ethylene diamine tetraacetic acid, washed twice with 0.5% BSA and then stained with PE-conjugated mouse anti-human CD29 (β_1_-integrin chain) antibody for 30 minutes. The stained cells were washed twice with 0.5% BSA and then fixed in cold ethanol (70%) for 30 minutes. Fixed cells were washed and analyzed with a fluorescence-activated cell sorter (FACS; Calibur, Becton-Dickinson, Franklin Lakes, NJ, USA), and the data were analyzed using a mod-fit β_1_-integrin expression analysis program.

### siRNA and Transfection

For siRNA transfection, the cells at 70–80% confluence were transfected with the designated siRNA at various concentrations (10–40 nM) for 48 hrs using the RNAimax transfection kit (Invitrogen, Carlsbad, CA, USA) and then stimulated with 17β-estradiol or exposed to flow.

### Statistical analysis

Results are expressed as mean ± SEM. Statistical analysis was performed by using an independent Student's *t* test for two groups of data and analysis of variance (ANOVA) followed by Scheffe's test for multiple comparisons. A *p* value of less than .05 was considered significant.

## Results

### Estrogen augments shear stress–induced ERK and p38 MAPK phosphorylations in MG63 cells

**Fig. 1:**
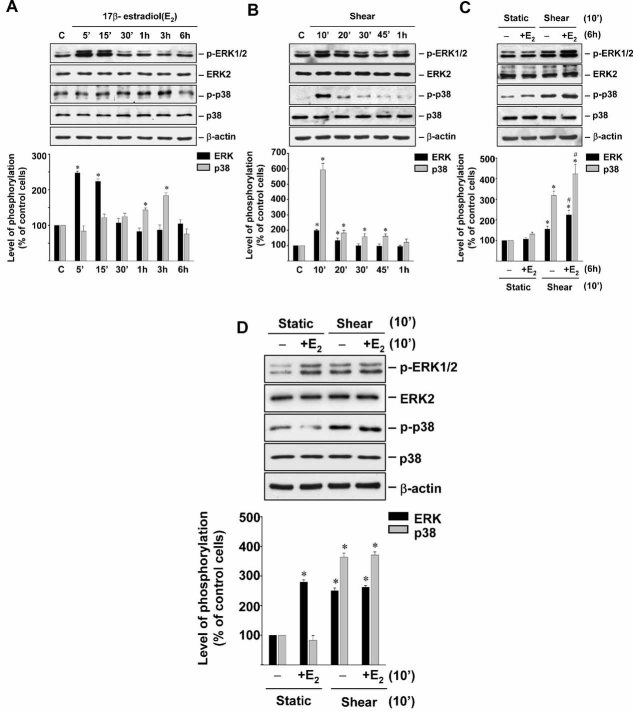
Estrogen augments shear stress–induced ERK and p38 MAPK phosphorylations in MG63 cells. MG63 cells were kept as controls or stimulated with 17β-estradiol (E_2_; 10 nM) (*A*) or shear stress (12 dyn/cm^2^) (*B*) for the times indicated, and their ERK and p38 MAPK phosphorylations were determined by using Western blot analysis. (*C*) Cells were treated with 17β-estradiol for 6 hours and then kept in static condition or exposed to shear stress for 10 minutes. (*D*) Cells were subjected to simultaneous stimuli with shear stress and 17β-estradiol for 10 minutes in the absence of 17β-estradiol pretreatment. The amounts of phosphorylated ERK and p38 MAPK in stimulated cells are presented as band densities (normalized to the total protein levels) relative to those in unstimulated control cells. The results are mean ± SEM from three independent experiments. *p < .05 versus unstimulated control cells. ^#^*p* < .05 versus sheared cells without 17β-estradiol pretreatment (*C*).

We first examined the individual effects of estrogen and shear stress on the activations of ERK and p38 MAPK in MG63 cells. MG63 cells were stimulated with 17β-estradiol (10 nM) for 5, 15, and 30 minutes and 1, 3, and 6 hours, and their phosphorylations of ERK and p38 MAPK were determined by Western blot analysis. As shown in Figure 1*A*, stimulation of MG63 cells with 17β-estradiol induced a rapid increase (significant within 5 minutes) in the ERK phosphorylation in these cells. In contrast, the increase in p38 MAPK phosphorylation induced by 17β-estradiol was later (significant within 1 hour). These increased levels of ERK and p38 MAPK phosphorylations decreased to nearly the basal levels 30 minutes and 6 hrs, respectively, after 17β-estradiol stimulation. Application of shear stress (12 dyn/cm^2^) to MG63 cells induced a rapid increase (significant within 10 minutes) in ERK and p38 MAPK phosphorylations in comparison with static controls (see 1*B*). These increased levels of ERK and p38 MAPK phosphorylations decreased to nearly the basal levels 30 minutes and 1 hour, respectively, after exposure to flow.

To investigate the synergistic effect of estrogen and shear stress on the ERK and p38 MAPK activations, MG63 cells were treated with 17β-estradiol for 6 hrs and then exposed to flow for 10 minutes in the presence of 17β-estradiol. The activations of ERK and p38 MAPK in these cells were compared with those in the cells stimulated with 17β-estradiol and shear stress alone. As shown in 1*C*, pretreatment of MG63 cells with 17β-estradiol before exposure to shear stress resulted in higher levels of ERK and p38 MAPK phosphorylations in these cells than in the cells exposed to shear stress alone. These results suggest that pretreatment of MG63 cells with estrogen before shearing augments the shear stress–induced ERK and p38 MAPK phosphorylations in these cells. This augmented effect of estrogen on shear stress–induced ERK and p38 MAPK phosphorylations was not seen in cells exposed to simultaneous stimuli with shear stress and 17β-estradiol for 10 minutes in the absence of 17β-estradiol pretreatment (see 1*D*).

### Estrogen induces β_1_-integrin expression in MG63 cells through ERs

**Fig. 2:**
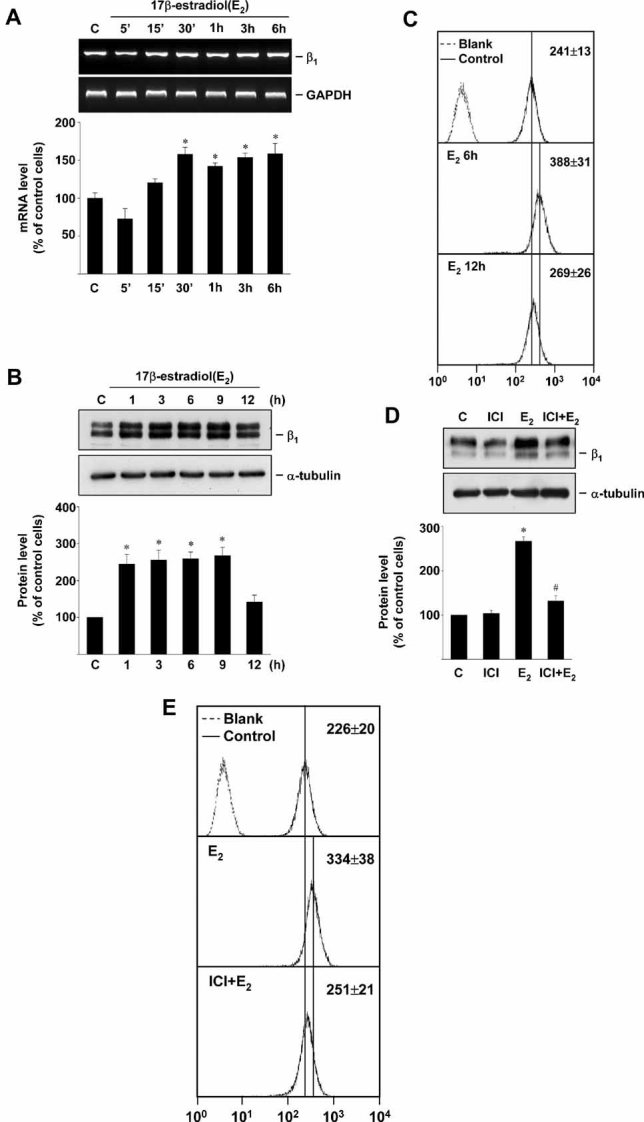
Estrogen induces β_1_-integrin expression in MG63 cells through ERs. MG63 cells were kept as controls or stimulated with 17β-estradiol (E_2_; 10 nM) for the times indicated (*A*–*C*). In some experiments, MG63 cells were kept as controls (C) or pretreated with ICI 182,780 (ICI; 1 µM) for 1 hr and then stimulated with 17β-estradiol (E_2_; 10 nM) for 6 hrs (ICI + E_2_) (*D*, *E*). The β_1_ mRNA and protein expressions were determined by using RT-PCR (*A*), Western blot (*B*, *D*), and flow cytometric (*C*, *E*) analyses, respectively. The results are shown as mean ± SEM from three separate experiments. Data in panels *A*, *B*, and *D* are presented as percentage changes in band density from unstimulated control cells and normalized to GAPDH (*A*) or α-tubulin protein level (*B*, *D*). In panels *C* and *E*, MG63 cells incubated with FITC-conjugated antibody alone were used as blanks. Numbers are mean ± SEM of mean fluorescent intensity for all experiments determined by comparison with corresponding blanks. *p < .05 versus unstimulated control cells. ^#^*p* < .05 versus E_2_-treated cells (*D*).

To investigate the effect of estrogen on β_1_-integrin expression in MG63 cells, MG63 cells were treated with 17 β-estradiol (10 nM) for 5, 15, and 30 minutes and 1, 3, and 6 hrs, and their mRNA expression was examined by RT-PCR. As shown in Figure 2*A*, the mRNA levels of β_1_-integrin were induced by 17β-estradiol at 30 minutes and remained elevated over the 6 hr period tested. The results of Western blot analysis showed that treatment of MG63 cells with 17β-estradiol induces β_1_-integrin protein expression within 1 hr of stimulation (see 2*B*). These increased levels of β_1_-integrin protein remained for 9 hrs and then declined to the basal levels 12 hrs after stimulation. The estrogen-induced increases in β_1_-integrin expression were confirmed by flow cytometric analysis, which showed that stimulation of MG63 cells with 17β-estradiol for 6 hours resulted in an increase in β_1_-integrin surface expression, with a mean fluorescence intensity of 388 compared with 241 in untreated cells, and that the β_1_-integrin decreased to near the control level (see 2*C*), in agreement with the Western blot result in 2*B*. These results indicate that estrogen induces gene and protein expressions of β_1_-integrin in MG63 cells, which is accompanied by the increase in β_1_-integrin expression on the cell surface.

To investigate whether ERs are required for estrogen-induced β_1_-integrin expression in MG63 cells, MG63 cells were pretreated with ICI 182,780 (1 µM), a specific ER antagonist, for 1 hour and then stimulated with 17β-estradiol (10 nM) for 6 hrs. The MG63 cell expression of β_1_-integrin was examined by both Western blot and flow cytometric analyses. As shown in 2*D*, stimulation of MG63 cells with 17β-estradiol for 6 hrs induced their β_1_-integrin expression. Pretreatment of MG63 cells with ICI 182,780 before 17β-estradiol stimulation inhibited the 17β-estradiol-induced β_1_-integrin expression. As a control, ICI 182,780 did not have effect on the basal levels of β_1_-integrin expression in MG63 cells. The inhibition in 17β-estradiol-induced β_1_-integrin expression by ICI 182,780 was confirmed by flow cytometric analysis, which showed that stimulation with 17β-estradiol resulted in an increase in β_1_-integrin protein expression on MG63 cell surface, with a mean fluorescence intensity of 334 compared with 226 in control unstimulated cells (see 2*E*); pretreating MG63 cells with ICI 182,780 reduced 17β-estradiol-induced β_1_-integrin expression to a mean fluorescence intensity of 251. These results indicate that ERs are required for estrogen induction of β_1_-integrin expression in MG63 cells.

### Estrogen-mediated augmentation of shear stress–induced ERK and p38 MAPK activations in MG63 cells is mediated by ERs through β_1_-integrin

**Fig. 3:**
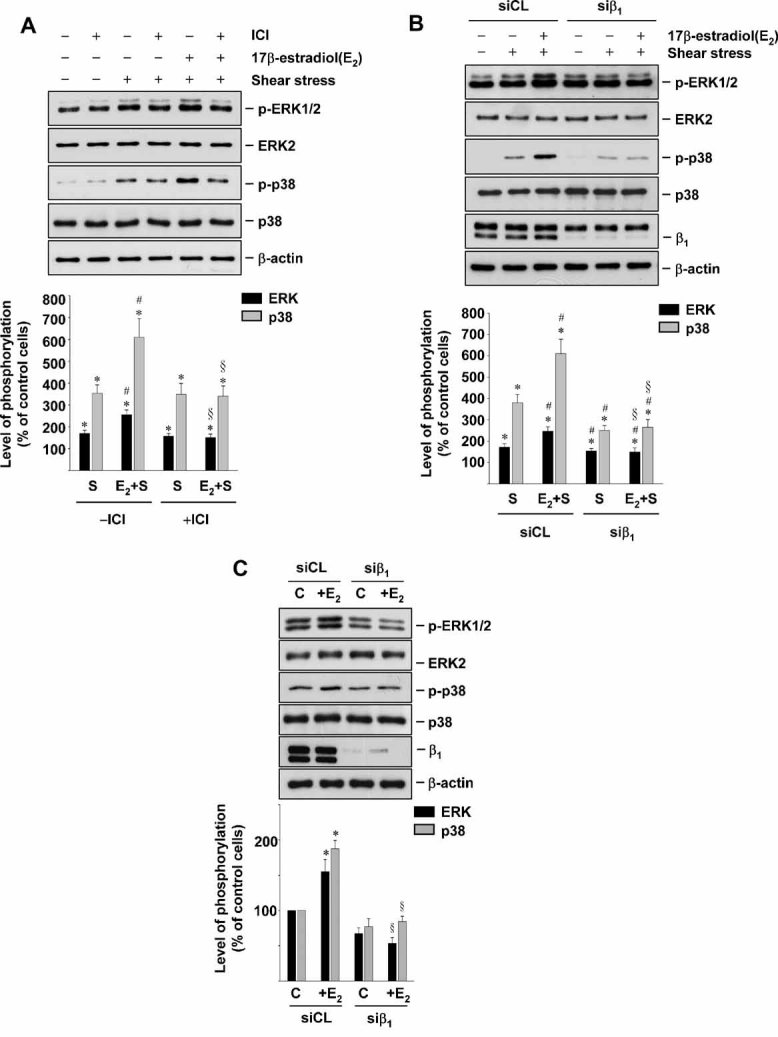
Estrogen-mediated augmentation of shear stress–induced ERK and p38 MAPK activations in MG63 cells is regulated by ERs and β_1_-integrin. MG63 cells were kept as controls or pretreated with ICI 182,780 (ICI; 1 µM) for 1 hr and then stimulated with 17β-estradiol (E_2_; 10 nM) for 6 hrs, followed by exposure to shear stress (S; 12 dyn/cm^2^) for 10 minutes (E_2_ + S) (*A*). In parallel experiments, MG63 cells were transfected with control (siCL) or β_1_-integrin-specific siRNA (siβ_1_) for 48 hours prior to 17β-estradiol and shear stress stimuli (*B*). (*C*) Cells were transfected with control or β_1_-integrin-specific siRNA for 48 hrs and then kept as controls or treated with 17β-estradiol for 15 minutes (for ERK) or 1 hr (for p38 MAPK). The phosphorylations of ERK and p38 MAPK in these cells were determined by Western blot analysis. Data are presented as the amounts (band densities normalized to the total protein levels) of phosphorylated ERK and p38 MAPK proteins relative to those in static control cells pretreated with vehicle control (*A*) or transfected with control siRNA (*B*, *C*) and are shown as mean ± SEM from three independent experiments. *p < .05 versus unstimulated control cells. ^#^*p* < .05 versus sheared cells without 17β-estradiol pretreatment. ^§^*p* < .05 versus 17β-estradiol/shear stress–stimulated cells pretreated with vehicle control (*A*) or transfected with control siRNA (*B*, *C*).

Given our findings that estrogen augments shear stress–induced ERK and p38 MAPK activations in MG63 cells and that estrogen induces β_1_-integrin expression through ERs, we investigated whether the augmented effect of estrogen on shear stress–induced ERK and p38 MAPK activations is mediated by ERs through β_1_-integrin. To this end, MG63 cells were kept as controls or pretreated with ICI 182,780 for 1 hr and then stimulated with 17β-estradiol for 6 hours, followed by exposure to shear stress at 12 dyn/cm^2^ for 10 minutes. As shown in Figure 3*A*, treatment of MG63 cells with 17β-estradiol before shearing augmented shear stress–induced ERK and p38 MAPK phosphorylations in MG63 cells compared with cells exposed to flow in the absence of 17β-estradiol pretreatment. Pretreating MG63 cells with ICI 182,780 before 17β-estradiol and shear stress stimuli inhibited this augmented effect of 17β-estradiol on shear stress–induced ERK and p38 MAPK phosphorylations but did not inhibit the increases in ERK and p38 MAPK phosphorylations induced by shear stress alone. Transfection of MG63 cells with β_1_-specific siRNA (40 nM, compared with control siRNA), which almost totally abolished the β_1_-integrin protein expression, inhibited the shear stress–induced ERK and p38 MAPK phosphorylations and the augmented effect of 17β-estradiol on shear activations of these MAPKs in MG63 cells (see 3*B*). Transfecting with β_1_-specific siRNA also inhibited the 17β-estradiol-induced ERK and p38 MAPK phosphorylations in MG63 cells (see 3*C*). Taken together, these results suggest that (1) the ERK and p38 MAPK activations in MG63 cells induced by shear stress alone are mediated by β_1_-integrin but not ERs, and (2) the augmented effect of estrogen on shear-induced ERK and p38 MAPK activations are mediated by both ERs and β_1_-integrin.

### Estrogen augments shear stress–induced *c-fos* and *Cox-2* expressions in MG63 cells through ERK and p38 MAPK pathways

MG63 cells were kept as controls or exposed to shear stress (12 dyn/cm^2^) for 15 and 30 minutes and and 1 hr, and their expression of *c-fos* and *Cox-2* genes was examined by quantitative real-time PCR. Exposure of MG63 cells to shear stress induced *c-fos* and *Cox-2* mRNA expressions (4*A*) within 15 minutes. For 1 hr of testing, the longer the duration of shearing, the greater was the increase in *Cox-2* mRNA expression. In contrast, the increased expression of *c-fos* mRNA declined but still elevated after 1 hr of flow application. Pretreating MG63 cells with specific inhibitor of either ERK (e.g., PD98059; 30 µM) or p38 MAPK (i.e., SB203580; 10 µM) or the combination of both inhibitors resulted in inhibitions, at least in part, in shear stress–induced *c-fos* and *Cox-2* expressions (see 4*B*), indicating that both ERK and p38 are involved in shear stress inductions of these two bone-formation-related genes in MG63 cells. The expressions of *c-fos* and *Cox-2* in MG63 cells were induced by treating with 17β-estradiol alone within 30 minutes (4*C*). These increased levels of *c-fos* and *Cox-2* mRNAs were declined to the basal levels 3 hours after 17β-estradiol stimulation.

**Fig. 4:**
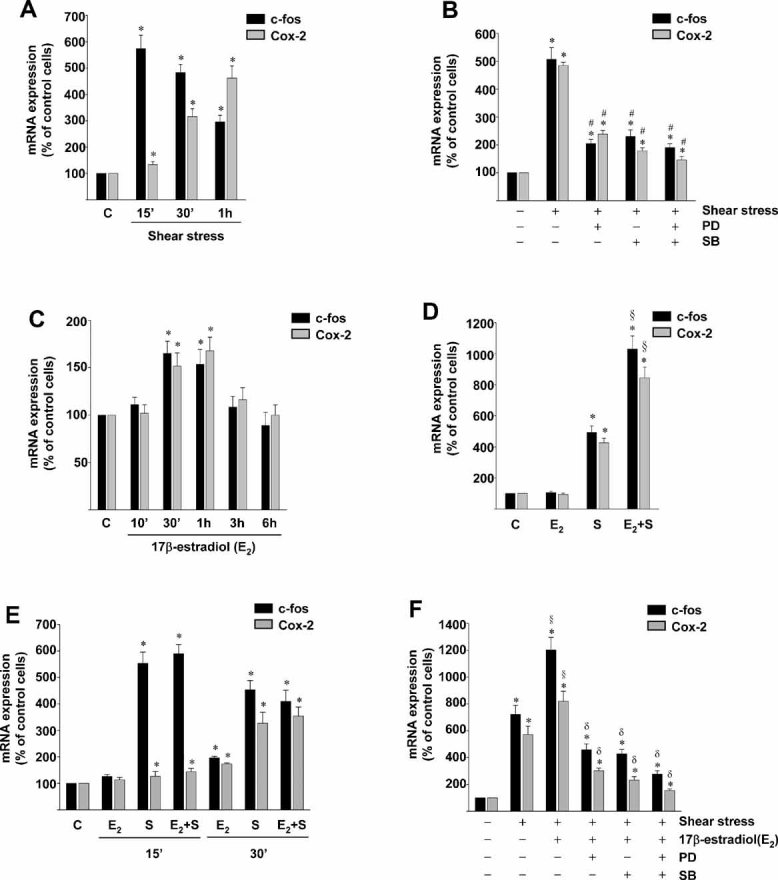
Estrogen augments shear stress–induced *c-fos* and *Cox-2* expressions in MG63 cells. MG63 cells were kept as controls (C), exposed to shear stress at 12 dyn/cm^2^ (S) (*A*), or stimulated with 17β-estradiol (E_2_; 10 nM) (*C*) for the times indicated, and their *c-fos* and *Cox-2* mRNA expressions were determined by quantitative real-time PCR. In some experiments, MG63 cells were pretreated with vehicle control DMSO or specific inhibitor of ERK (PD98058; 30 µM) or p38 MAPK (SB203580; 10 µM) or a combination of these inhibitors for 1 hr before exposure to shear stress for 15 minutes (for *c-fos*) or 1 hour (for *Cox-2*) (*B*). In parallel experiments, the cells were stimulated with 17β-estradiol for 6 hrs and then exposed to shear stress for 15 minutes (for *c-fos*) or 1 hour (for *Cox-2*) (E_2_ + S) (*D*). Prior to 17β-estradiol and shear stress treatments, the cells were preincubated with PD98058 or SB203580 or a combination of these inhibitors for 1 hour (*F*). (*E*) MG63 cells were subjected to simultaneous stimuli with shear stress and 17β-estradiol for 15 and 30 minutes without 17β-estradiol pretreatment. Data are presented as percentage changes relative to the unstimulated control cells (normalized to β-actin gene expression) and shown as mean + SEM from three or four separate experiments. *p < .05 versus unstimulated control cells. ^#^*p* < .05 versus sheared cells without inhibitor pretreatment (*B*). ^§^*p* < .05 versus sheared cells without 17β-estradiol pretreatment (*D*, *F*). ^δ^*p* < .05 versus sheared/E_2_-treated cells without inhibitor pretreatment (*F*).

To investigate the synergistic effect of estrogen and shear stress on the expressions of *c-fos* and *Cox-2*, MG63 cells were treated with 17β-estradiol for 6 hrs and then exposed to flow for 15 minutes (for *c-fos*) or 1 hr (for *Cox-2*). Pretreatment of MG63 cells with 17β-estradiol before shearing resulted in an augmentation of shear stress–induced *c-fos* and *Cox-2* expressions compared with cells exposed to flow in the absence of 17β-estradiol pretreatment (4*D*). However, this estrogen augmentation of shear stress–induced *c-fos* and *Cox-2* expressions was not seen in cells exposed to simultaneous stimuli with shear stress and 17β-estradiol for 15 and 30 minutes in the absence of 17β-estradiol pretreatment (see 4*E*). Pretreating MG63 cells with either PD98059 (30 µM) or SB203580 (10 µM) or a combination of these inhibitors abolished the estrogen augmentation of shear stress–induced *c-fos* and *Cox-2* expressions (see 4*F*), suggesting that the augmented effect of estrogen on shear induction of *c-fos* and *Cox-2* in MG63 cells is mediated by the ERK and p38 MAPK pathways.

### The estrogen augmention of shear stress–induced *c-fos* and *Cox-2* expressions is mediated by ERs through β_1_-integrin

MG63 cells were kept as controls or pretreated with ICI 182,780 for 1 hr and then stimulated with 17β-estradiol for 6 hrs, followed by exposure to shear stress at 12 dyn/cm^2^ for 15 minutes (for *c-fos*) or 1 hour (for *Cox-2*). The expressions of the *c-fos* and *Cox-2* genes in these cells were examined by quantitative real-time PCR. Application of shear stress to MG63 cells induced their *c-fos* and *Cox-2* mRNA expressions (5*A*). These shear stress–induced expressions of mRNAs were augmented by pretreating the cells with 17β-estradiol before shearing. Pretreating MG63 cells with ICI 182,780 before 17β-estradiol and shear stress stimuli resulted in inhibitions in the augmented effect of 17β-estradiol on shear inductions of *c-fos* and *Cox-2* but did not affect the induction of these genes by shear stress (as compared with the application of shear stress alone). In parallel experiments, MG63 cells were transfected with control or β_1_-specific siRNA (40 nM) and then treated with 17β-estradiol for 6 hours, followed by exposure to shear stress for 15 minutes (for *c-fos*) or 1 hour (for *Cox-2*). As shown in 5*B*, transfection of MG63 cells with β_1_-specific siRNA (compared with control siRNA) inhibited not only the shear inductions of *c-fos* and *Cox-2* but also the augmentation effect of 17β-estradiol on shear inductions of these genes. Taken together with our findings that estrogen induces β_1_-integrin expression through ERs, these results suggest that the augmentation effect of estrogen on shear inductions of *c-fos* and *Cox-2* in MG63 cells is mediated by ERs through β_1_-intergrin.

**Fig. 5:**
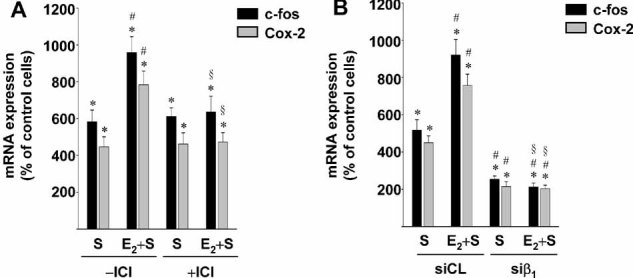
Estrogen-mediated augmentation of shear stress–induced *c-fos* and *Cox-2* expressions is mediated by both ERs and β_1_-integrin. MG63 cells were kept as controls or pretreated with ICI 182,780 (ICI; 1 µM) for 1 hr (*A*) and then stimulated with 17β-estradiol (E_2_; 10 nM) for 6 hours, followed by exposure to shear stress (S; 12 dyn/cm^2^) for 15 minutes (for *c-fos*) or 1 hour (for *Cox-2*) (*A*). In parallel experiments, MG63 cells were transfected with control (siCL) or specific siRNA of β_1_-integrin (siβ_1_) for 48 hours prior to 17β-estradiol and shear stress stimuli (*B*). The expressions of *c-fos* and *Cox-2* mRNA were determined by quantitative real-time PCR. Data are presented as the expression (normalized to the β-actin gene expression) of *c-fos* or *Cox-2* relative to those in static control cells pretreated with vehicle control (*A*) or transfected with control siRNA (*B*) and are shown as mean ± SEM from three independent experiments. *p < .05 versus unstimulated control cells. ^#^*p* < .05 versus sheared cells without 17β-estradiol pretreatment. ^§^*p* < .05 versus 17β-estradiol/shear stress–stimulated cells pretreated with vehicle control (*A*) or transfected with control siRNA (*B*).

### Confirmation of the augmented effect of estrogen on shear-induced signaling and gene expression in primary HOBs

**Fig. 6:**
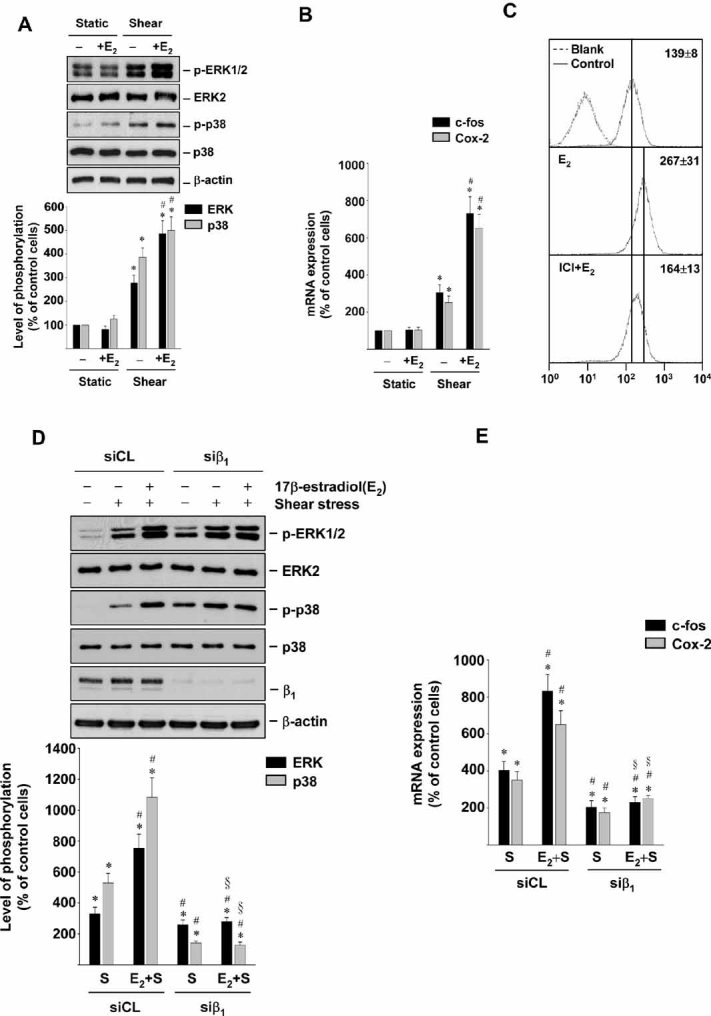
Confirmation on the augmented effect of estrogen on shear stress–induced signaling and gene expression in primary HOBs. Primary HOBs were kept as controls or stimulated with 17β-estradiol (E_2_; 10 nM) for 6 hrs and then exposed to shear stress at 12 dyn/cm^2^ for 10 minutes (for MAPK) (*A*), 15 minutes (for *c-fos*), or 1 hr (for *Cox-2*) (*B*). The phosphorylations of ERK and p38 MAPK (*A*) and expressions of *c-fos* and *Cox-2* (*B*) in these cells were determined by using Western blot and quantitative real-time PCR, respectively. In parallel experiments, HOBs were transfected with control (siCL) or specific siRNA (40 nM) of β_1_-integrin (siβ_1_) for 48 hrs prior to 17β-estradiol and/or shear stress stimuli (*D*, *E*). (*C*) Flow cytometric analysis of β_1_-integrin surface expression on HOBs treated with 17β-estradiol for 6 hrs. In some experiments, HOBs were pretreated with ICI 182,780 (ICI; 1 µM) for 1 hr prior to 17β-estradiol stimulation. HOBs incubated with FITC-conjugated antibody alone were used as blanks. Data in panels *A*, *B*, *D*, and *E* are presented as percentage changes relative to the unstimulated control cells [normalized to the MAPK total protein levels (*A*, *D*) or β-actin gene expression (*B*, *E*)] and shown as mean ± SEM from three separate experiments. Numbers in panel *C* are mean ± SEM of mean fluorescent intensity for three experiments determined by comparison with corresponding blanks. *p < .05 versus unstimulated control cells. ^#^*p* < .05 versus sheared cells without 17β-estradiol pretreatment. ^§^*p* < .05 versus 17β-estradiol/shear stress–stimulated cells transfected with control siRNA (*D*, *E*).

Since MG63 cells are a highly transformed cell line derived originally from an osteogenic sarcoma (despite sharing some properties characterized for osteoblasts), we examined whether similar results on the effect of estrogen on shear-induced signaling and gene expression in MG63 cells can be obtained with primary HOBs. As shown in Figure 6, HOBs pretreated with 17β-estradiol for 6 hours with subsequent exposure to shear stress had higher levels of ERK and p38 MAPK phosphorylations (see 6*A*) and *c-fos* and *Cox-2* expressions (see 6*B*) than the cells exposed to shear stress alone. Treating HOBs with 17β-estradiol for 6 hours resulted in an increase in β_1_-integrin surface expression; this estrogen induction of β_1_-integrin was inhibited by pretreating the cells with ICI 182,780 (see 6*C*). Moreover, transfecting HOBs with β_1_-specific siRNA (compared with control siRNA; 40 nM for each) resulted in inhibitions, at least in part, in shear-induced ERK and p38 MAPK phosphorylations (see 6*D*) and *c-fos* and *Cox-2* expressions (see 6*E*), as well as the 17β-estradiol-mediated augmentation of these shear-induced MAPK signaling and gene expressions in these cells. However, in contrast to MG63 cells, HOBs transfected with β_1_-specific siRNA had higher basal levels of MAPK signaling and *c-fos* and *Cox-2* expressions than the control siRNA-transfected cells. These results confirm the role of estrogen in modulating shear stress–induced signaling and gene expression through ER induction of β_1_-integrin in primary HOBs.

## Discussion

The aim of this study was to investigate the synergistic role of estrogen and shear stress in signal transduction and gene expression in human osteoblast-like MG63 cells and primary osteoblasts using activations of ERK and p38 MAPK and expressions of *c-fos* and *Cox-2* as readouts. Through a series of systematic studies, we have demonstrated that estrogen augments the shear stress–induced ERK and p38 MAPK activations and *c-fos* and *Cox-2* expressions in osteoblast-like cells through ER-mediated increases in β_1_-integrin expression. Several lines of evidence support this conclusion. First, MG63 cells treated with 17β-estradiol for 6 hrs followed by exposure to shear stress had higher levels of ERK and p38 MAPK phosphorylations and *c-fos* and *Cox-2* expressions than the cells exposed only to shear stress without 17β-estradiol pretreatment. Second, MG63 cells stimulated with 17β-estradiol for 6 hours had higher levels of β_1_-integrin mRNA and protein expressions than the unstimulated cells. Blockage of ERs by the ER-specific antagonist ICI 182,780 inhibited the 17β-estradiol-induced β_1_-integrin expression in MG63 cells. Third, pretreating MG63 cells with ICI 182,780 or transfecting the cells with β_1_-specific siRNA inhibited the augmentation effects of 17β-estradiol on shear stress–induced ERK and p38 MAPK phosphorylations and *c-fos* and *Cox-2* expressions in MG63 cells. Fourth, similar results on the augmented effect of estrogen on MAPK signaling and *c-fos* and *Cox-2* expressions were obtained using HOBs. Our findings provide insights into the mechanism by which estrogen and shear stress play synergistic roles in modulating signaling and gene expression in osteoblast-like cells.

Both estrogen and mechanical forces have been shown to be positive regulators for osteoblast proliferation and bone formation.(22–25) However, the synergistic roles of these two stimulators in modulating signaling and gene expression in osteoblasts remain unclear. Jagger and colleagues(26) demonstrated that estrogen administration augments compressive force–induced osteogenic response in rat caudal vertebrae. Joldersma and colleagues(27) showed that estrogen enhances shear stress–induced production of prostaglandin E_2_ (PGE_2_) by osteoblasts derived from postmenopausal and nonosteoporotic women. Bakker and colleagues(28) showed that estrogen and shear stress exert additive effects on the productions of PGE_2_ and nitric oxide by bone cells derived from osteoporotic women (aged 62–90 years). These results suggest that estrogen may affect the signaling and function of bone cells in response to mechanical forces, which consequently may affect the mechanoresponsiveness of bone in the process of bone formation and remodeling. In this study, we demonstrated for the first time that pretreatment of osteoblast-like MG63 cells and HOBs with estrogen augments the shear stress–induced ERK and p38 MAPK phosphorylations and *c-fos* and *Cox-2* expressions. These results indicate that estrogen may regulate mechanosensitivity of bone cells via the MAPK pathway, which may result in the increased expression of bone-formation-related genes, for example, *c-fos* and *Cox-2*, and the consequent modulation in bone formation and remodeling in response to mechanical stimuli.

Several studies have indicated that ERs may play important roles in bone formation and adaptation in response to mechanical forces.(29–31) Lee and colleagues(29) applied mechanical strain to the ulnae of *ERα* knockout (*ERα*^−/−^) mice and found that the strain-induced bone formation of cortical bone in *ERα*^−/−^ mice is only one-third of that in *ERα*^+/+^ littermates. They further demonstrated that osteoblast-like cells derived from *ERα*^−/−^ mice have deficient response to mechanical strain.(30) Damien and colleagues (31) showed that mechanical strain stimulates osteoblast proliferation through ERs in males as well as females. Aguirre and colleagues (32) demonstrated that the ER antagonist ICI 182,780 abrogates ERK activation induced by cyclic stretch in osteocytic cells and that *ERα* and *ERβ* expressions are required for ERK activation by cyclic stretch in osteoblastic and osteocytic cells. These results have been interpreted to suggest that ERs may serve as mechanosensors to play significant roles in modulating bone cell signaling and function and hence bone formation in response to mechanical stimuli. Using antibodies against phosphorylated ER-α, our recent studies did show that application of shear stress at 12 dyn/cm^2^ to MG63 cells induces their ER-α phosphorylation (data not shown). However, this study shows that blockage of ERs by ICI 182,780 did not inhibit shear stress–induced ERK and p38 MAPK phosphorylations and *c-fos* and *Cox-2* expressions in MG63 cells, indicating that ERs may not be involved in the effect of shear stress per se on MAPK signaling and gene expression in MG63 cells. The detailed mechanisms underlying the discrepancy in biologic effects of ERs on mechanical responses of cells between our present results and previous studies remain unclear. Recent studies have indicated that different patterns of mechanical force application (e.g., steady versus oscillatory patterns of shear forces) exert differential effects on cell signaling, gene expression, and function through different molecular mechanisms.(33) Whether different types of mechanical loading, such as the steady fluid shear stress used in this study and cyclic stretch/strain used in other reports, induce signaling and gene expression in cells through different mechanisms remain to be determined. Our present results show that blockage of ERs by ICI 182,780 resulted in a reduction in the augmentation effects of estrogen on shear stress–induced ERK and p38 MAPK phosphorylations and *c-fox* and *Cox-2* expressions in MG63 cells, suggesting that ERs are involved in the estrogen-induced augmentation of shear stress responsiveness of signal transduction and gene expression in osteoblast-like cells.

Integrins play significant roles in mechanical responses of cells on the ECM. A recent study by Plotkin and colleagues(34) demonstrated that the effect of mechanical stretch on osteocytic cells can be transmitted by integrins, which are required for mechanical stretch–induced activation of ERK that plays important roles against cell apoptosis induced by different stimuli. In vascular endothelial cells, estrogen has been shown to induce β_1_-integrin expression and hence cell attachment and migration, which consequently may promote neovascularization and vessel repair.(19) This may be the reason for the observation that women prior to menopause have a lower cardiovascular risk but a higher incidence of several blood vessel–related autoimmune diseases, including Takayasu arteritis and systemic lupus erythematosus, than men.(35–37) The results of our present study showed that estrogen can induce the mRNA and protein expressions of β_1_-integrin in osteoblast-like MG63 cells and HOBs, in addition to endothelial cells. This estrogen-induced expression of β_1_-integrin requires ERs and contributes to the augmentation effects of estrogen on shear stress–induced ERK and p38 MAPK signaling and *c-fos* and *Cox-2* expressions in these osteoblast-like cells and HOBs. These results suggest that β_1_-integrin plays an important role in the modulation of signaling and gene expression in osteoblastic cells in response to the synergistic interaction of chemical and mechanical stimuli, for example, estrogen and shear stress.

In this study, shear stress was applied to the cells at the time point where the effects of estrogen on MAPK signaling and *c-fos* and *Cox-2* gene expressions have subsided (i.e., 6 hours of 17β-estradiol pretreatment before shearing). This treatment schedule allows us to clarify whether estrogen could exert effects on shear stress–induced signaling and gene expression even though its individual effect has disappeared. To elucidate whether the effect of shear stress still could be enhanced by estrogen applied together with shear stress without pretreatment, MG63 cells were subjected to shear stress in the presence of 17β-estradiol for 10 minutes (for MAPK) and 15 and 30 minutes (for *c-fos* and *Cox-2*), and the results showed that simultaneous stimuli with shear stress and estrogen did not result in an augmentation in shear stress–induced MAPK signaling (see 1*D*) and gene expression (see 4*E*) in MG63 cells compared with cells exposed to shear stress alone. The lack of an augmented effect of estrogen on shear stress–induced signaling and gene expression in these experiments could be due to the short exposure times (i.e., 10, 15, and 30 minutes), where the β_1_-integrin protein expression has not been induced by estrogen.

The proto-oncogene *c-fos* has been shown to be involved in mitogenic responses of osteoblastic cells.(38) Stable expression of *c-fos* in mice has been demonstrated in developing bones, cartilages, and teeth.(39) Overexpression of *c-fos* in transgenic and chimeric mice enhances bone and cartilage development.(40) In contrast, homozygous *c-fos*^−/−^ mice are growth-retarded and develop osteopetrosis, with deficiencies in bone remodeling and tooth eruption, compared with normal littermates.(41) Recent studies have demonstrated that estrogen induces *c-fos* expression in rat bone.(42) In addition, mechanically induced bone formation has been shown to be preceded by the expression of *c-fos* in the whole animal.(43) These results suggest that c-fos protein may serve as a critical molecule for bone formation and development and that *c-fos* expression may be affected by estrogen and mechanical forces in vivo. Cox-2 is a rate-limiting enzyme in the conversion of membrane-related arachidonic acid to prostaglandins to mediate the stimulatory effects of estrogen and mechanical loading on bone formation.(44,45) *Cox-2*^–/–^ mice have decreased bone density in comparison with normal littermates.(46) Bending forces applied to the tibiae of rats were found to increase *Cox-2* and *c-fos* mRNA expressions in vivo.(46) These reports suggest that the expressions of *c-fos* and *Cox-2* may play significant roles in estrogen- and mechanical loading–induced bone formation and remodeling. In this study, we demonstrated that both estrogen and mechanical forces can induce *c-fos* and *Cox-2* expressions in osteoblast-like cells. Our study further demonstrated that estrogen and shear stress can exert additive effects on the induction of *c-fos* and *Cox-2* expressions in osteoblast-like cells, suggesting that estrogen and mechanical forces may play synergistic roles in the process of bone formation and remodeling.

Deficiency in estrogen has been shown to be a risk factor to induce osteoporosis in postmenopausal women. Recent studies demonstrated that osteoblasts derived from osteoporotic patients showed less adhesive ability and lower levels of integrin/adhesion-mediated signaling activation than the cells derived from nonosteoporotic patients.(47) It is possible that the estrogen-less condition in postmenopausal women may result in a reduction in mechanical sensitivity of osteoblasts by decreasing the expression of integrins and the activation of integrin/adhesion-mediated signaling events, which consequently may influence the formation and remodeling of bone in response to mechanical stimuli.

In summary, this study demonstrated for the first time that estrogen augments the shear stress–induced MAPK signaling and *c-fos* and *Cox-2* expressions in human osteoblast-like MG63 cells and primary osteoblasts. This estrogen-induced augmentation of signaling and gene expression in response to shear stress is mediated by ERs through increased expression of β_1_-integrin. The identification of this synergistic role of estrogen and shear stress in modulating signaling and gene expression in osteoblasic cells not only provides new insights into the mechanism by which chemical stimuli may influence mechanical responses of bone cells but also aids in the future development of bone tissue engineering and pharmacologic therapies to increase bone formation.
